# Frank’s Sign in Polycystic Ovary Syndrome and Sideburn Adiposity: A Case Report and Illustrated Hypothesis

**DOI:** 10.7759/cureus.15744

**Published:** 2021-06-18

**Authors:** Mohammed Abrahim

**Affiliations:** 1 Family Medicine, McMaster University, Hamilton, CAN

**Keywords:** diagonal earlobe crease. frank’s sign, visceral fat depot, polycystic ovary syndrome (pcos), facial fat, metabolic diseases, clinical case report, physical exam, clinical features, facial plastic, head and neck and endocrine

## Abstract

The diagnosis of polycystic ovary syndrome (PCOS) remains uniquely dependent on physical examination signs. Diagonal earlobe crease (DELC) also referred to as “Frank’s sign, has been associated with several cardiometabolic disorders with further investigations reporting that its occurrence was more prevalent among aging men and thought to a sign of aging skin. However, there is no clear mechanism of development or rationale behind such associations. The author is reporting a novel association between Frank's sign and PCOS in a 23-year-old female patient. The presence of the DELC in young women could alert clinicians to the need to further investigate a diagnosis of PCOS. The author is providing an accompanying hypothesis to suggest possible common pathogenesis that links the two to visceral adiposity of the face.

## Introduction

Polycystic ovarian syndrome (PCOS) is the most common endocrinopathy among women in reproductive years [1]. The diagnostic criteria are based on the presence of at least two of the following three criteria: history of chronic anovulation, polycystic ovaries on pelvic ultrasound, and clinical or biological features of hyperandrogenism [1]. PCOS commonly presents during adolescence, therefore early diagnosis is crucial, when suspicion arises, through the screening of physical signs to prevent and treat its metabolic and psychological comorbidities [1].

The diagonal earlobe crease (DELC) was first described in 1973 by Dr. S. T. Frank and was observed to be associated with coronary artery disease among some patients [2]. In the following years, the DELC was named “Frank’s sign”, with further investigations reporting that its occurrence was more prevalent among aging men and its aetiology was suggested to be merely a crease of aging skin [3]. Over the past five decades, numerous studies have reported a plethora of associations between DELC and several cardiometabolic disorders [4]. Concurrently, visceral obesity is an established risk factor for cardiometabolic diseases, with a growing body of research demonstrating a potential causal association [5]. Similarly, visceral obesity is strongly associated with PCOS, with increasing evidence demonstrating the involvement of visceral adipose tissue (VAT) in PCOS pathogenesis [6]. We report herein the association between PCOS, DELC, and facial visceral adiposity in a young female patient in addition to an explanatory anatomical hypothesis.

## Case presentation

The author reports a case of bilateral DELC in a 23-year-old white female patient with PCOS. PCOS diagnosis was based on her menstrual irregularity and hirsutism since adolescence. She also had PCOS-associated metabolic disorders such as type 2 diabetes, nonalcoholic fatty liver disease, dyslipidemia, and hypothyroidism. She didn’t suffer from any cardiovascular diseases. In terms of lifestyle risk factors, the patient did not smoke tobacco or consume alcohol. Her activity levels were within the normal range. She also indicated that there was a strong family history of type 2 diabetes. Her medications included metformin, sodium-glucose co-transporter 2 (SGLT2) inhibitors, and levothyroxine. 

**Figure 1:**
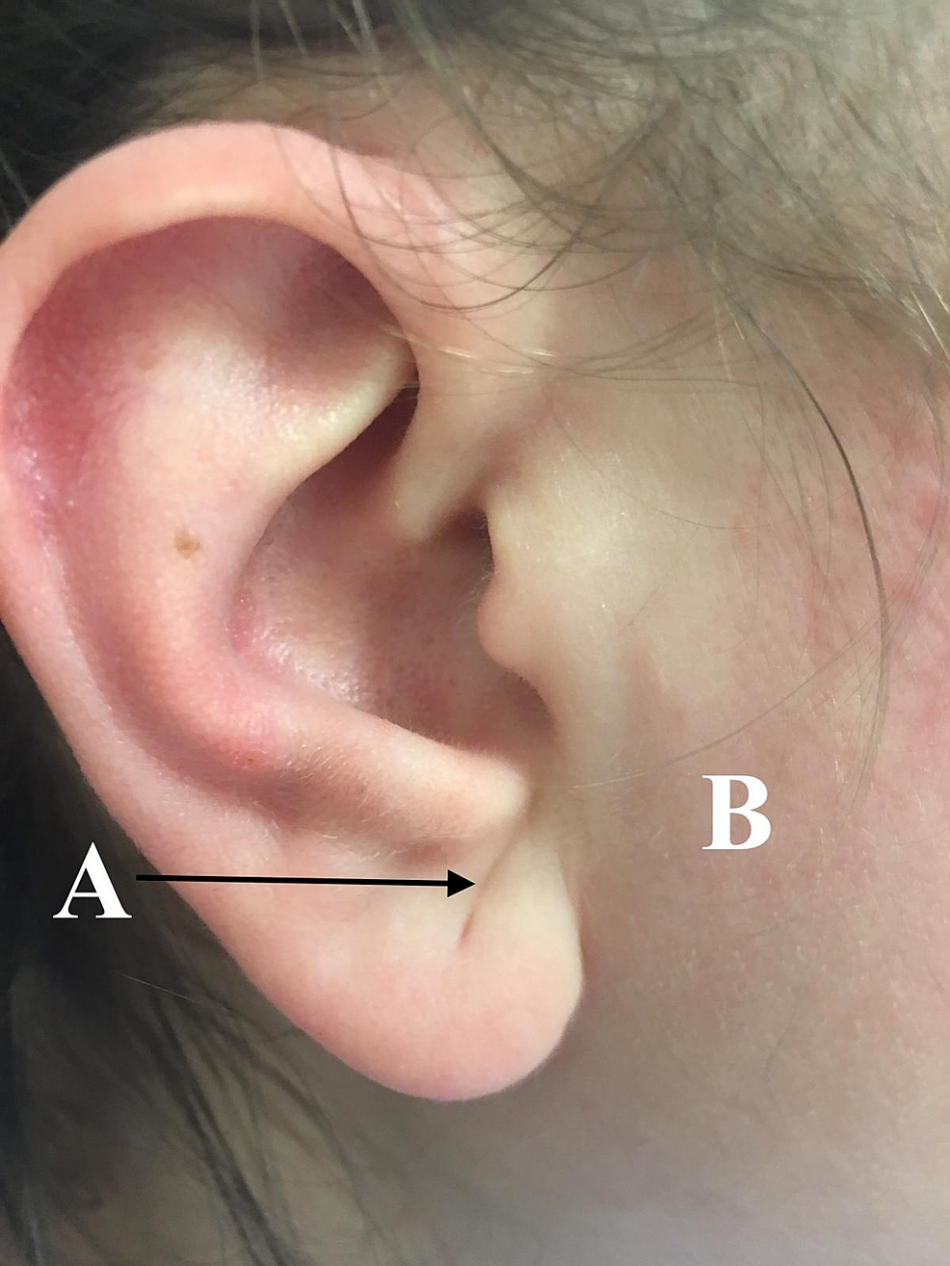
Diagonal earlobe crease and PCOS. The diagonal earlobe crease (point: A) and facial obesity of the sideburn region (point: B). PCOS: polycystic ovarian syndrome.

The physical examination revealed that the patient had an android-type phenotype with centripetal fat distribution within the trunk and the face. However, she had no features suggestive of Cushing's syndrome. Bodyweight was 84 kilograms with height recorded as 162 centimeters; this gave the patient a BMI of 32.0 and a waist circumference of 92 centimeters. No acanthosis nigricans or striae were noted. Normal thyroid gland on palpation was also noted and the cardiovascular and pulmonary physical exams were unremarkable. An abdominal exam revealed a triangular male-pattern distribution of hair in the lower abdomen. It was also noted that the patient appeared to have bilateral symmetrical diagonal earlobe creases (Figure 1, point A) and localized areas of lateral facial fat corresponding to the buccal fat pad (BFP) of the sideburn regions of the cheeks (Figure 1, point B). 

 

## Discussion

The reported association between DELC and PCOS could be explained as linked to visceral obesity. Visceral fat depots are not limited to the abdomen, they also occur in the face within the BFP [7]. Despite being anatomically separate, the BFP and abdominal visceral adipose tissue appear to be histologically and metabolically identical and, therefore, the BFP is occasionally referred to as visceral fat of the face [7]. BFP is located between the muscles of mastication, with its surface anatomy corresponding to the sideburn region of the lateral face [7]. BFP is connected to the auricle with Loré’s fascia which is a tympano-parotid band running from the parotid gland to the intertragal incisura of the auricular cartilage to the depth of the tympanomastoid fissure of the skull [8].

Furthermore, the size of abdominal visceral adipose tissue has been found to be strongly associated with the size of buccal adipose tissue [9]. This relation appears to be specific to visceral fat rather than general adiposity [10]. Therefore it is hypothesized that visceral adiposity could be the underlying pathogenesis of both DELC and the metabolic syndrome in PCOS. The mechanism of development of DELC could be hypothesized as a consequence of visceral obesity of the face which increases the size of BFP displacing the parotid gland laterally against the bony attachment of Loré’s fascia in the skull. Consequently, the expanding soft tissues of the lateral face encroach on the site of Loré’s fascia attachment as an anchor leading to the folding of the earlobe creating the characteristic DELC as illustrated anatomically in Video 1.

**Video 1:** Diagonal earlobe creases (Frank’s sign) anatomical explanation. Folding of the earlobe secondary to sideburn facial obesity.

## Conclusions

To our knowledge, this is the first case to report the association of DELC and PCOS. Furthermore, our case demonstrates that DELC does occur in young women with no signs of skin aging. Additionally, the author is suggesting that facial visceral adiposity, particularly that which corresponds to the sideburn regions of the face, could be the mechanical driver initiating internal folding of the earlobe that creates the characteristic DELC.

The author invites clinicians to be mindful of the presence of Frank’s sign among their younger patient populations, particularly those exhibiting symptoms of PCOS. Should DELC be found to be associated with PCOS, the physical sign has potential utility in the latter’s diagnosis. Further research is needed to assess the incidence and prevalence of DELC within the general population and confirm the potential association described above. The mechanical effect of facial visceral adiposity, as the underlying mechanism linking DELC and PCOS, also requires further investigation.
